# HIV-1 Nef Targets HDAC6 to Assure Viral Production and Virus Infection

**DOI:** 10.3389/fmicb.2019.02437

**Published:** 2019-10-30

**Authors:** Sara Marrero-Hernández, Daniel Márquez-Arce, Romina Cabrera-Rodríguez, Judith Estévez-Herrera, Silvia Pérez-Yanes, Jonathan Barroso-González, Ricardo Madrid, José-David Machado, Julià Blanco, Agustín Valenzuela-Fernández

**Affiliations:** ^1^Laboratorio de Inmunología Celular y Viral, Unidad de Farmacología, Sección de Medicina, Facultad de Medicina, Universidad de La Laguna (ULL), La Laguna, Spain; ^2^Unidad Virología y Microbiología del IUETSPC, Universidad de La Laguna (ULL), La Laguna, Spain; ^3^BioAssays SL, Campus de Cantoblanco, Madrid, Spain; ^4^Departmento de Genética, Fisiología y Microbiología, Facultad de Biología, Universidad Complutense de Madrid (UCM), Madrid, Spain; ^5^AIDS Research Institute IrsiCaixa, Institut de Recerca en Ciències de la Salut Germans Trias i Pujol (IGTP), Badalona, Spain; ^6^Universitat de Vic-Central de Catalunya, UVIC-UCC, Catalonia, Spain

**Keywords:** HIV-1, Nef, HDAC6, Pr55Gag, acidic-endocytic/lysosomal degradation, autophagy, infection, viral production

## Abstract

HIV Nef is a central auxiliary protein in HIV infection and pathogenesis. Our results indicate that HDAC6 promotes the aggresome/autophagic degradation of the viral polyprotein Pr55Gag to inhibit HIV-1 production. Nef counteracts this antiviral activity of HDAC6 by inducing its degradation and subsequently stabilizing Pr55Gag and Vif viral proteins. Nef appears to neutralize HDAC6 by an acidic/endosomal-lysosomal processing and does not need the downregulation function, since data obtained with the non-associated cell-surface Nef-G2A mutant – the cytoplasmic location of HDAC6 – together with studies with chemical inhibitors and other Nef mutants, point to this direction. Hence, the polyproline rich region P72xxP75 (69–77 aa) and the di-Leucin motif in the Nef-ExxxLL160-165 sequence of Nef, appear to be responsible for HDAC6 clearance and, therefore, required for this novel Nef proviral function. Nef and Nef-G2A co-immunoprecipitate with HDAC6, whereas the Nef-PPAA mutant showed a reduced interaction with the anti-HIV-1 enzyme. Thus, the P72xxP75 motif appears to be responsible, directly or indirectly, for the interaction of Nef with HDAC6. Remarkably, by neutralizing HDAC6, Nef assures Pr55Gag location and aggregation at plasma membrane, as observed by TIRFM, promotes viral egress, and enhances the infectivity of viral particles. Consequently, our results suggest that HDAC6 acts as an anti-HIV-1 restriction factor, limiting viral production and infection by targeting Pr55Gag and Vif. This function is counteracted by functional HIV-1 Nef, in order to assure viral production and infection capacities. The interplay between HIV-1 Nef and cellular HDAC6 may determine viral infection and pathogenesis, representing both molecules as key targets to battling HIV.

## Introduction

Human immunodeficiency virus type 1 (HIV-1) has developed multiple strategies to evade the immune system and to establish a persistent and chronic infection (Moir et al., 2011; Towers and Noursadeghi, 2014; Pyndiah et al., 2015; Sauter and Kirchhoff, 2016; Sumner et al., 2017). The viral accessory proteins of HIV-1 are responsible for deploying several of these strategies, which overall ensure virus infection and survival (Pyndiah et al., 2015; Sumner et al., 2017). The negative regulatory factor, Nef, is one of these auxiliary proteins with a decisive role in viral replication and pathogenesis (Kestler et al., 1991; Foster and Garcia, 2007; Gorry et al., 2007; Kirchhoff et al., 2008). HIV-1-Nef is a myristoylated protein (206 amino acids; 27–35 kDa) that is expressed in the early phase of HIV-1 infection (Guy et al., 1987; Foster et al., 2011), being highly conserved among primate lentiviruses HIV-1, HIV-2, and SIV (Stevenson, 1996; Renkema and Saksela, 2000). Because of the over-lapping effector domains on it to interact with multiple cellular proteins, this small viral protein is functionally complex. Hence, Nef fosters a favorable environment for viral replication, subverting a plethora of host cell factors and functions (Arold et al., 1997; O’Neill et al., 2006; Kirchhoff et al., 2008; Lindwasser et al., 2008; Noviello et al., 2008; Foster et al., 2011). In fact, the ability of Nef to internalize and/or downregulate membrane receptors is integral to the effects and key functions it has during infection. Nef promotes either degradation or intracellular sequestration of host receptors, including restriction factors and immune cell receptors, thereby facilitating the escape of HIV-1 from the immune responses (Sugden et al., 2016). By hijacking the adaptor proteins (AP)-1, AP-2, and AP-3 involved in membrane trafficking, Nef alters the intracellular distribution of different membrane receptors and affects their stability. For example, Nef captures AP-1 to facilitate the endocytosis and sequestration of major histocompatibility complex type I (MHC-I) molecules (Roeth et al., 2004; Jia et al., 2012; Pawlak and Dikeakos, 2015; Dirk et al., 2016), limiting recognition of infected cells by the immune system (Collins et al., 1998). In parallel, the ability of Nef to bind p56Lck, AP-2, and AP-3 promotes the endocytosis and degradation of CD4, in CD4+ T-cells, which limits superinfection, antibody-dependent cell-mediated cytotoxicity, and viral egress (Schwartz et al., 1995; Piguet et al., 1999; Ross et al., 1999; Michel et al., 2005; Wildum et al., 2006; Chaudhuri et al., 2007; Pham et al., 2014; Ren et al., 2014; Veillette et al., 2014). Recently, it has been described that Nef targets CD28, an immune co-stimulatory receptor, driving its lysosomal degradation. Thus, the ability of infected cells to respond to CD28-mediated stimulation would depend on the amount of intracellular Nef, maybe helping in the mechanism of HIV-1 latency (Pawlak et al., 2018).

However, the involvement of Nef in HIV-1 pathogenesis, its role in triggering viral infection, and in the development of AIDS is not mechanistically well understood (Kestler et al., 1991; Deacon et al., 1995; Kirchhoff et al., 1995; Foster and Garcia, 2007). Although incorporated in the viral particle, Nef’s key functions during viral replication rather concerns its early expression upon viral infection (Laguette et al., 2009). Some on the inhibition of lysosome- and proteasome-associated degradative activities, together with the use of different Nef mutants, suggest that Nef-mediated enhancement of infectivity requires alteration of protein stability and/or membrane trafficking, as well as cell-signaling pathways (Goldsmith et al., 1995; Madrid et al., 2005; Wei et al., 2005; Coleman et al., 2006). So far, two cellular factors have been mainly involved in Nef-mediated enhancement of virion infectivity: dynamin 2 and myeloid-restricted tyrosine kinase p59Hck (Saksela et al., 1995; Briggs et al., 2001; Madrid et al., 2005; Pizzato et al., 2007). Nef also targets the host transmembrane, serine incorporator 5 protein (SERINC5), impairing its incorporation into nascent virions and its antiviral activity at a post-fusion step, where SERINC5 may abrogate some events required for the translocation of the viral core into the cytoplasm (Rosa et al., 2015; Trautz et al., 2016). Nef targets SERINC5 by two distinct events, impairing its endosomal targeting and promoting its internalization from the cell-surface (Trautz et al., 2016). SERINC3 also has anti-HIV-1 activity, with Nef as its viral antagonist (Fackler, 2015; Usami et al., 2015). These Nef actions occur early upon infection of permissive cells, and is related to the CD4 downregulation function (reviewed in Pereira and daSilva, 2016).

Although the fact that Nef stabilizes HIV-1 Gag at the plasma membrane, that it facilitates cell-to-cell viral transfer (Malbec et al., 2013), and its further processing have been reported on, little is known about the role of Nef in viral production rate (Costa et al., 2004; Mendonca et al., 2014). Our previous studies indicate that the inhibition of the functional cytoplasmic enzyme, histone deacetylase 6 (HDAC6), significantly enhances HIV-1 replication in primary peripheral blood lymphocytes (Valenzuela-Fernandez et al., 2005, 2008). Moreover, HDAC6 regulates infectivity of nascent HIV-1 virions by interacting with APOBEC3G (A3G; Apolipoprotein B mRNA-editing enzyme-catalytic, polypeptide-like 3G), stabilizing it and promoting the autophagic degradation of Vif, thereby impairing the incorporation of Vif in nascent viral particles (Valera et al., 2015). Recently, we have reported a direct correlation between the inability of primary HIV-1 envelope complexes (Envs) to signal through CD4 and to infect, and the natural control of the HIV-1 infection in a cluster of long-term non-progressor, elite controllers (LTNP-EC)’ individuals (Casado et al., 2018). These Envs are unable to bind CD4 with high affinity and to signal stabilizing acetylated α-tubulin, correlating these facts with the low fusion, infection, and replication activities of viruses from this LTNP-EC cluster. Hence, HIV-1 Envs that cannot inhibit HDAC6-tubulin deacetylase antiviral activity are not infectious, whereas HIV-1 Envs able to signal through CD4 to overcome HDAC6-deacetylase activity stabilize acetylated α-tubulin and are therefore infectious and pathogenic, in viremic non-progressor, progressor and rapid progressor patients (Casado et al., 2018; Cabrera-Rodriguez et al., 2019). Therefore, HDAC6 represents a new antiviral factor capable of determining viral infection and disease progression in HIV+ individuals. In this context, we aim to seek for a potential interplay between HDAC6 and Nef, which could be key in the control of HIV-1 viral production and infection.

In this work, we present new data that indicate that HDAC6 inhibit HIV-1 production by promoting the degradation of the viral polyprotein Pr55Gag. Remarkably, Nef counteracts this HDAC6 activity by associating with the enzyme and inducing its degradation. Nef appears to target HDAC6 by an acidic/endosomal-lysosomal processing independently of its effects on protein downregulation. This new Nef proviral function stabilizes Pr55Gag and Vif viral proteins, and assures Pr55Gag location and aggregation at plasma membrane, viral egress, and the infectivity of viral particles. Therefore, it is plausible to consider HDAC6 as an anti-HIV restriction factor neutralized by Nef to foster a favorable environment for viral production and infection.

## Materials and Methods

### Antibodies and Reagents

Rabbit anti-HDAC6 (H-300; sc-11420), rabbit anti-GFP (FL; sc-8334), and rabbit anti-HA-probe (Y-11; sc-805), polyclonal antibodies (polyAbs), and mouse monoclonal antibodies (mAbs), anti-HIV-1-Nef (sc-65906), anti-HIV-1-Vif (319; sc-69731), anti-GFP (B-2; sc-9996), anti-p62/SQSTM1 (D-3; sc-28359), and anti-HA-probe (F-7; sc-7392), and Polybrene (sc-134220) were obtained from Santa Cruz Biotechnology (Santa Cruz, CA, United States). Rabbit anti-(HIV1 p55 + p24 + p17) (ab63917) polyAb was from Abcam (Cambridge Science Park, Cambridge, United Kingdom). The neutralizing mAb RPA-T4 (eBioscience, San Diego, CA, United States) directed against CD4 was phycoerythrin (PE)-labeled (for flow cytometry). Mabs anti-α-tubulin (T6074) and anti-acetylated α-tubulin (T7451); Z-Leu- Leu-Leu-al or MG132 (C2211), 3-Methyladenine (M9281), E-64d (E8640), Pepstatin A (77170) and Bafilomycin A1 Ready Made Solution (SML 1661) inhibitors, and secondary horseradish peroxidase (HRP)-conjugated Abs, specific for any Ab species assayed were purchased from Sigma-Aldrich (Sigma-Aldrich, St. Louis, MO, United States), and secondary Alexa Fluor 568-labeled goat-anti-mouse Ab was from Molecular Probes (Eugene, OR, United States). Complete^TM^ Protease Inhibitor Cocktail (11697498001) was obtained from Roche Diagnostics (GmbH, Mannheim, Germany).

### DNA Plasmids and Viral DNA Constructs

**FIGURE 1:**
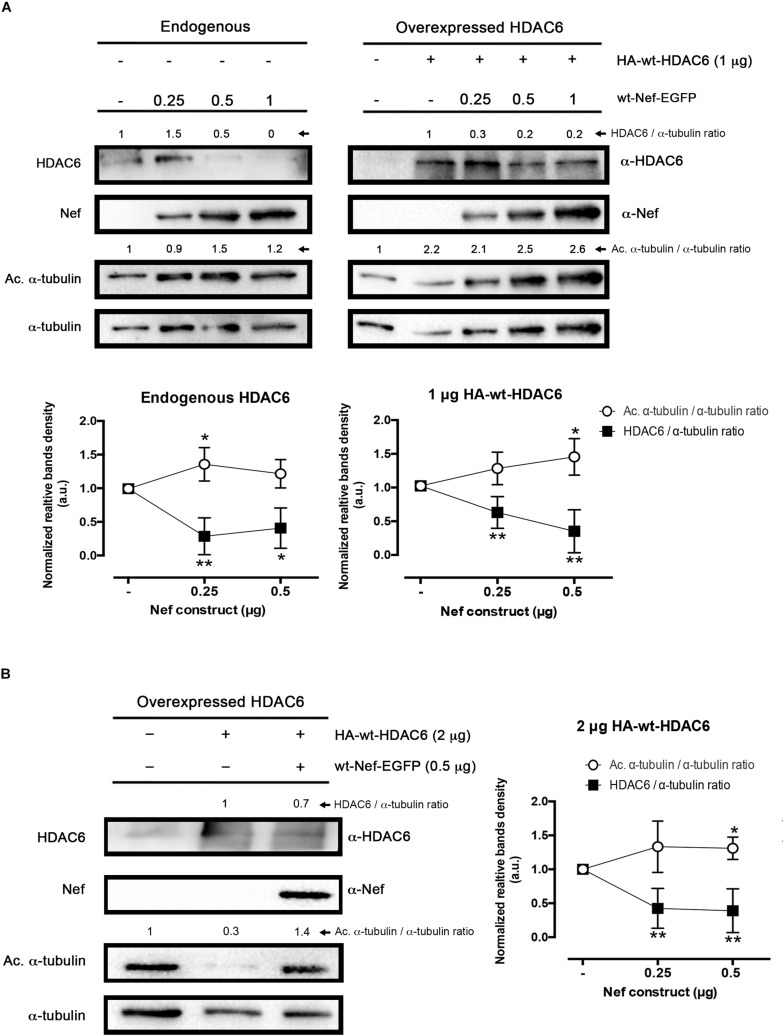
Effect of HIV-1 Nef on HDAC6 degradation. **(A)** Quantitative western blot analysis of wt-Nef-EGFP, dose-response degradative effects on endogenous HDAC6 (*left*) and over-expressed HA-wt-HDAC6 (1 μg; *right*). Acetylated α-tubulin/α-tubulin and HDAC6/α-tubulin intensity band ratios are shown, where enhancement of acetylated α-tubulin levels is a read-out for loss of HDAC6-deacetylase activity. A representative western blot performed in HEK-293T cells is shown. Histograms show intensity bands quantitation of top western blots for wt-Nef-EGFP-mediated HDAC6 degradation for the endogenous and the over-expressed enzyme, normalized by the total amount α-tubulin, under any experimental condition. Data are means ± standard errors of the means (SEM) of six independent experiments, in the case of endogenous HDAC6, and nine independent experiments for over-expressed HDAC6 (1 μg). **(B)** Quantitative western blot analysis of the efficiency of an intermediate concentration of Nef (0.5 μg) to degrade a high amount of HA-wt-HDAC6 (2 μg-cDNA). Acetylated α-tubulin/α-tubulin and HDAC6/α-tubulin ratios are shown, and α-tubulin is the control for total protein. Acetylated α-tubulin levels observed are a read-out for loss of HDAC6-deacetylase activity. Histograms show intensity bands quantitation of western blot for wt-Nef-EGFP-mediated over-expressed HDAC6 degradation, normalized by the total amount α-tubulin, under this experimental condition. Data are expressed as mean ± S.E.M. of nine independent experiments. In **(A,B)**, acetylated α-tubulin/α-tubulin and HDAC6/α-tubulin ratios are shown in arbitrary light units (a.u.). When indicated, ^∗^*P* < 0.05 and ^∗∗^*P* < 0.01 are *P-*values for Student’s *t-*test.

Vectors for expression of full-length wild-type (wt) or mutated HIV-1Lai-Nef were constructed in the pRcCMV plasmid, and Nef mutants (Nef-G2A; Nef-E160A, Nef-EA; Nef-LL164-5/AA, Nef-LLAA; and Nef-P72xxP75/A, Nef-PPAA) were generated by PCR-directed mutagenesis using appropriate primers as described (Erdtmann et al., 2000; Madrid et al., 2005). Vectors for expression of wt or mutated Nef-GFP were constructed in the pEGFP as described (Greenberg et al., 1998). Nef-CFP expression plasmids were constructed in the pECFP-N1 by PCR and cloning in frame to CFP using *Eco*RI/*Bam*HI restriction sites. HDAC6 construct was provided by Drs. X.-J. Yang and N. R. Bertos (Molecular Oncology Group, Department of Medicine, McGill University Health Centre, Montreal, QC, Canada) (Bertos et al., 2004; Valenzuela-Fernandez et al., 2005; Valera et al., 2015). When indicated, these plasmids were cloned into pEGFP-C1, pDSRED2 (using *Age*I/*Not*I restrictions sites) (Clontech, Palo Alto, CA, United States) or N-terminal tagged with the terminal influenza hemagglutinin (HA) epitope, as reported (Madrid et al., 2005; Valenzuela-Fernandez et al., 2005; Valera et al., 2015). The pcDNA3.1 (Life Technologies) or pEGFP-C1/ECFP-C1 (Clontech, Palo Alto, CA) vectors were used as a control of cDNA transfection or to express free EGFP/ECFP. The pNL4-3.Luc.R-E- provirus (Δ*nef*/Δ*env*) and the R5.tropic BaL.01-envelope (*env*) glycoprotein plasmid were obtained via the NIH AIDS Research and Reference Reagent Programme (catalog numbers 6070013 and 11445, respectively). The pGag-EGFP vector (catalog no. 11468; from Marilyn Resh), allowing imaging of intracellular and cell-surface aggregated Gag in live cells, which directs Rev-independent expression of an HIV-1-Gag-EGFP fusion protein (Schwartz et al., 1992), were obtained through the NIH AIDS Research and Reference Reagent Program, and used as we previously reported (Garcia-Exposito et al., 2011). In this study, in general, we will use these working plasmids amounts: for Nef 0.5 μg cDNA, and 1 μg cDNA for HDAC6, as in Figure 1A.

### Cells

The human CEM.NKR-CCR5 permissive cell line (catalog number 4376, NIH AIDS Research and Reference Reagent Program) and the HEK-293T cells (catalog number 103, NIH AIDS Research and Reference Reagent Program) were grown at 37°C in a humidified atmosphere with 5% CO_2_ in RPMI 1640 medium (Lonza, Verviers, Belgium) in the case of the CEM.NKR-CCR5 cells, and in DMEM (Lonza) in the case of HEK-293T, both medium supplemented with 10% fetal calf serum (Lonza), 1% L-glutamine, and 1% penicillin-streptomycin antibiotics (Lonza) and mycoplasma free (Mycozap antibiotics, Lonza), and both cell lines were regularly split every 2–3 days. The HEK-293T cell line was cultured to 50–70% confluence in fresh supplemented medium 24 h before cell transfection with viral or human DNA constructs.

### Western Blotting

Protein expression was determined by SDS-PAGE and Western blotting in cell lysates. HEK-293T cells co-transfected with different cDNA constructs using linear polyethylenimine, with an average molecular mass of 25 kDa (PEI25K) (Polyscience, Warrington, PA, United States) dissolved in 150 mM NaCl. A mixture of PEI25k/plasmids (3:1 ratio (wt/wt) was gently vortexed, incubated for 20–30 min at room temperature (RT), and then added to cells in culture. Briefly, 48 h after transfection, cells were lysated in lysis buffer (1% Triton-X100, 50 mM Tris-HCl pH 7,5, 150 mM NaCl, 0.5% sodium deoxycholate, and protease inhibitor (Roche Diagnostics), for 30 min and sonicated for 30 s at 4°C. The effects of the different inhibitors were similarly assayed in HEK-293T cells. Twenty four hour post-transfection cells were treated for 5 h at 37°C with any of the following inhibitors: MG132, 20 μM dissolved in dimethyl sulfoxide (DMSO), to inhibit the proteasome; 3-MA, 5 mM in PBS, to inhibit autophagosome formation and subsequent autophagic degradation, monitored by detecting p62/SQSTM1 protein; or Bafilomicyn A1, 100 nM in DMSO, as a specific inhibitor of vacuolar-typeH+-ATPase, to inhibit acidification and protein degradation in lysosomes of cultured cells; and E-64d + PepsA, 10 μg/mL in DMSO, as protease inhibitors. Equivalent amounts of protein (40 μg), determined using the bicinchoninic acid (BCA) method (Millipore Corporation, Billerica, MA, United States), were resuspended and treated by Laemmli buffer and then were separated in 12% SDS-PAGE and electroblotted onto 0.45 μm polyvinylidene difluoride membranes (PVDF; Millipore) using Trans-blot Turbo (Bio-Rad, Hercules, CA, United States). Membranes were blocked 5% non-fat dry milk in TBST (100 mM Tris, 0.9% NaCl, pH 7.5, 0.1% Tween 200) for 30 min and then incubated with specific antibodies. Proteins were detected by luminescence using the ECL System (Bio-Rad), and analyzed using a ChemiDoc MP device and Image LabTM Software, Version 5.2 (Bio-Rad).

### Co-immunoprecipitation Assays

HEK-293T cells (3 × 10^5^ in a 6-well plates) were co-transfected with different plasmids using PEI25k to express the tagged proteins: HA-wt-HDAC6 (1 μg), wt-Nef-EGFP (0.5 μg), Nef-G2A-EGFP (0.5 μg), and Nef-PPAA-EGFP (0.5 μg). HA-wt-PI4P5-K Ia (0.5 μg) and pEGFPC1 (0.5 μg) plasmids were used as tag controls conditions. For HDAC6/Nef interaction, protein G magnetic beads (Millipore) were incubated with 2 μg of the different antibodies (anti-HA and anti-EGFP) for 2 h at RT and then were washed three times with cold PBS 0.1% Tween (Sigma-Aldrich) using a magnet. Cells were lysed and the proteins were quantified in the cleared lysates by the BCA assay. Cell lysates were incubated with the different antibodies and with 1 mg/mL RNAse A enzyme (Roche) overnight on a rotating wheel at 4°C. Beads were then washed three times and resuspended in Laemmli buffer. Bound proteins were analyzed by SDS-PAGE and western blotting together with the input cell fractions.

### Fluorescence Confocal Microscopy Assay

HEK-293T cells (3 × 10^5^ cells in sterile glass coverslips-Ø 12 mm) were co-transfected with the different plasmids (wt-HDAC6-EGFP (1 μg) and wt-Nef-ECFP (0.5 μg) using PEI25k, in order to analyze their co-distribution. Forty eight hours post-transfection, cells were washed three times with PBS, fixed for 20 min in 2% paraformaldehyde in PBS. Coverslips were mounted in Mowiol-antifade (Dako, Glostrup, Denmark) and image acquisition was performed by confocal microscopy (Leica TCS SP5; Leica Microsystems, Wetzlar, Germany). For high-resolution acquisition a 1.35 NA objective (60x) was used. The co-distribution of fluorescent HDAC6 and Nef proteins was line scan quantified using MetaMorph software (Universal Imaging, Downington, PA, United States), as we previously described (Barroso-Gonzalez et al., 2009a, b; Garcia-Exposito et al., 2011).

### Flow Cytometry Analysis

Nef effects on CD4 cell-surface expression in HDAC6-expressing permissive CEM.NKR-CCR5 cells was studied by flow cytometry analysis. Briefly, 24 h nucleofected cells with fluorescent constructs were incubated, in ice-cold PBS buffer, with an anti-CD4 antibody coupled to PE. Labeling of cell-surface receptors was performed by staining with a PE-conjugated IgG isotype. Cells were then washed by ice-cold PBS, fixed in PBS with 1% paraformaldehyde, and analyzed by flow cytometry (XL-MCL system; Beckman-Coulter, CA, United States), measuring cell-surface CD4 receptor labeling as similarly described (Valenzuela-Fernandez et al., 2005; Barrero-Villar et al., 2008, 2009; Barroso-Gonzalez et al., 2009a; Garcia-Exposito et al., 2013). Basal cell fluorescence intensity for CD4 labeling was determined by staining cells with a PE-conjugated IgG isotype control in cells over-expressing free EGFP/ECFP proteins, measured through argon single-laser excitation at 458 nm, and both detected in the FL1 channel. Flow cytometry data were analyzed by Flowing software 2.5.1 (Turku Centre for Biotechnology, University of Turku, Turku, Finland).

### Total Internal Reflection Fluorescence Microscopy (TIRFM) and Analysis of Gag Localization and Aggregation at Plasma Membrane

Living HEK-293T cells, transiently over-expressing Pr55Gag-EGFP, alone or with wt-Nef-DsRed and/or wt-HDAC6-ECFP, seeded in poli-D-Lysine coated coverslips were imaged at 48 h from transfection in chambers containing a Krebs-HEPES buffer with 2 mM Ca^2+^, with an inverted microscope Zeiss 200M (Zeiss, Jena, Germany) through a 1.45 NA objective (αFluar, 100×, Zeiss) using an immersion fluid (n488 = 1.518, Zeiss) and under TIRF illumination, as we similarly reported for HIV-1 and cellular studies (Barroso-Gonzalez et al., 2009a, b; Garcia-Exposito et al., 2011). The expanded beam of an argon ion laser (LASOS Lasertechnik, Jena, Germany) was band-pass filtered, aligned with precise angle measured as described (Barroso-Gonzalez et al., 2009a, b; Garcia-Exposito et al., 2011), and used to selectively excite fluorescent proteins only located near to plasma membranes. Each cell was imaged using HC Image acquisition software (Hamamatsu Photonics) with 0.25 s exposures by EM-CCD digital camera (C9100-13, Hamamatsu Photonics, Hamamatsu City, Japan). Epifluorescence images were taken using an Hg lamp that were projected onto a back-illuminated CCD camera (AxioCam MRm, Zeiss) through a dichroic and specific band-pass filter for fluorescent wt-Nef-DsRed and wt-HDAC6-ECFP. To quantify the degree of Pr55Gag-EGFP aggregation at plasma membrane, the TIRFM images were background subtracted using MetaMorph (Universal Imaging), and analyzed by plotting 3 lines of 15 μm-length along the cell diameter. Signal was scored positive when the fluorescence of the spots in the line scans were at least mean ± 2SD of the local background. Data were pooled in histograms that show averaged number of aggregates per cell.

### Production of Viral Particles

Pseudotyped HIV-1 viral particles were obtained as previously described (Valenzuela-Fernandez et al., 2005; Barrero-Villar et al., 2008, 2009; Barroso-Gonzalez et al., 2009a; Garcia-Exposito et al., 2011, 2013; Valera et al., 2015; Casado et al., 2018; Cabrera-Rodriguez et al., 2019). Briefly, replication-deficient viral particles were derived from the luciferase-expressing reporter virus HIV/Δ*nef*/Δ*env*/*luc*+ (in which the luciferase gene is inserted into the *nef* ORF and does not express the envelope glycoprotein) with an R5-tropic (BaL.01) glycoprotein *env* plasmid. R5-tropic HIV-1 viral particles were produced in 12-wells plates by co-transfecting HEK-293T packaging cells (70% confluence) with pNL4-3.Luc.R-E- (1 μg) and R5-tropic (BaL.01) Env-glycoprotein vector (1 μg) and also over-expressing different plasmid combinations of HDAC6 construct and/or Nef constructs. Viral plasmids were transduced in HEK-293T cells using X-tremeGENE HP DNA transfection reagent (Roche). After the addition of X-tremeGENE HP to the viral plasmids the solution was mixed in 100 μL of DMEM medium without serum or antibiotics, and incubated for 20 min at RT prior to adding it to HEK-293T cells. The cells were cultured for 48 h to allow viral production; after this time viral particles were harvested and HEK-293T cells were lysed to analyze the expression of the different proteins. Viral stocks were normalized by p24-Gag content as measured with an enzyme-linked immunosorbent assay test (GenscreenTM HIV-1 Ag Assay; Bio-Rad, Marnes-la-Coquette, France). Virions were used to infect CEM.NKR-CCR5 cells after ELISA-p24 quantification and normalization.

### Luciferase Viral Infection Assay

Untreated CEM.NKR-CCR5 cells (9 × 10^5^ cells in 24-well plates with 20 μg/mL of polybrene) were infected for 2 h with a synchronous dose of viral inputs (100 ng of p24), in a total volume of 1 mL RPMI 1640 (by centrifugation for 2 h at 335 g at 25°C), and for 4 h at 37°C, as described previously (Valenzuela-Fernandez et al., 2005; Barrero-Villar et al., 2008, 2009; Barroso-Gonzalez et al., 2009a; Garcia-Exposito et al., 2011, 2013; Valera et al., 2015; Casado et al., 2018; Cabrera-Rodriguez et al., 2019). Unbound virus was then removed by washing the infected cells, and 48 h after infection luciferase activity (associated to productive viral entry into infected cells) was measured using a Luciferase Assay System (Promega Corporation, Madison, WI, United States) and a microplate reader (VictorTM X5, PerkinElmer, Waltham, MA, United States). Data were analyzed using GraphPad Prism 6.0 software (GraphPad Software, San Diego, CA, United States).

## Results

### HIV-1 Nef Induces HDAC6 Degradation

To ascertain the ability of the Nef viral protein to overcome the anti-HIV-1 activity of HDAC6 (Valenzuela-Fernandez et al., 2005, 2008; Valera et al., 2015; Casado et al., 2018; Cabrera-Rodriguez et al., 2019), we first analyzed HDAC6 enzyme degradation by full-length recombinant HIV-1 Nef in HEK-293T cells (Figure 1). We observed that Nef degrades endogenous (Figure 1A, *left blot*) as well as over-expressed HDAC6 (Figure 1A, *right blot*, and Figure 1B), in a dose-dependent manner. This fact correlates well with the increase observed in the acetylation of α-tubulin (Figure 1A, *dot histograms*), a main substrate for the HDAC6-tubulin deacetylase enzyme (Hubbert et al., 2002; Valenzuela-Fernandez et al., 2005, 2008; Casado et al., 2018; Cabrera-Rodriguez et al., 2019). We further detected that Nef induced the degradation of a higher amount of over-expressed HDAC6, thereby confirming the efficiency of Nef to degrade HDAC6 (Figure 1B).

### Inhibitors Affecting Acidification of Organelles and Lysosomal Proteinases Impair HIV-1 Nef-Promoted HDAC6 Degradation

**FIGURE 2:**
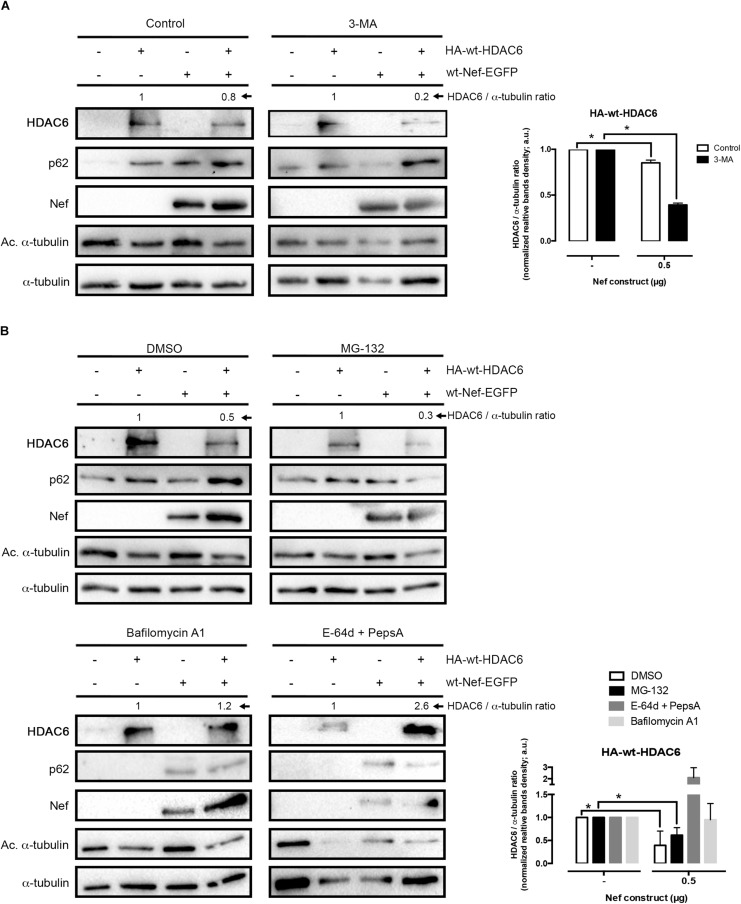
Nef targets HDAC6 by an acidic, endocytic/lysosomal degradative route. **(A)** Quantitative western blot analysis of Nef-mediated HDAC6 degradation in HEK-293T cells transfected with wt-Nef-EGFP (0.5 μg), HA-wt-HDAC6 (1 μg), and treated with the 3-MA inhibitor and the vehicle control (PBS). HDAC6/α-tubulin ratios are shown in arbitrary light units (a.u.). Histograms show quantification of western blot bands for the HDAC6/α-tubulin ratio in the absence of the presence of Nef, under inhibitor or control experimental condition. Data are mean ± S.E.M. of four independent experiments. ^∗^*P* < 0.05 value for Student’s *t*-test. **(B)** Quantitative western blots analysis of Nef-mediated HDAC6 degradation in HEK-293T cells transfected with wt-Nef-EGFP (0.5 μg), HA-wt-HDAC6 (1 μg), and treated with different chemical inhibitors, such as MG132, Bafilomycin A1 and “E-64d + PepsA” combination. DMSO is vehicle control. HDAC6/α-tubulin ratios are shown (a.u.). Histograms show quantification of the inhibitory effect exerted by Bafilomycin A1 and E-64d + PepsA on wt-Nef-EGFP-mediated HA-wt-HDAC6 degradation. Data are mean ± S.E.M. of five independent experiments in the case of MG132 inhibitor and two in the case of the Bafilomycin A1 and E-64d + PepsA inhibitors ^∗^*P* < 0.05 value for Student’s *t-*test.

We next aimed to pinpoint the mechanism involved in Nef-mediated degradation of HDAC6. For that purpose, we performed the same experiments above in the presence of some inhibitors for autophagy (3-methyladenine; 3-MA) and proteasome (MG132) degradative pathways, in HEK-293T cells. These chemical inhibitors were used as we previously reported (Valera et al., 2015) to inhibit aggresome formation (3-MA) and associated HDAC6-triggered autophagy degradation of target proteins (Seglen and Gordon, 1982; Liang et al., 1999; Klionsky et al., 2008a; Valera et al., 2015), and to inhibit the proteasome degradation route (MG132) (Valera et al., 2015). We observed that neither 3-MA nor MG132 altered the ability of Nef to degrade HDAC6 (Figures 2A,B). In control conditions, Nef triggers HDAC6 clearance (Figure 2A, *Control blot*), thus leading to an accumulation of p62/SQSTM1, which interacts and works with HDAC6 in the autophagic clearance of ubiquitinated protein aggregates, where p62 fades too (Bjorkoy et al., 2006; Pankiv et al., 2007; Valera et al., 2015; Yan et al., 2019). Because Nef-mediated HDAC6 degradation increases acetylation of α-tubulin and stabilizes p62 (Figure 2A, *Control blot*), Nef is not recruiting HDAC6 to the aggresome/autophagy degradative pathway. MG132-mediated inhibition of proteasome degradation has been described to indirectly favor autophagy, due to the cytoplasmic accumulation of polyubiquitinated chains, derived from polyubiquitinated proteins non-degraded by the proteasome, which triggers this optional degradative pathway (Hao et al., 2013), as we also reported (Valera et al., 2015). In this matter, we monitored low levels of p62 protein in MG132 treated cells compared to control, vehicle-treated cells (Figure 2B, *MG132 blot*), being indicative of a more active autophagic pathway, even under Nef-mediated HDAC6 degradative experimental conditions. On the contrary, in control cells treated with DMSO, Nef promotes HDAC6 degradation while p62 is stabilized. Indeed, there is an increase in the level of p62 in Nef-expressing cells (Figure 2B, *DMSO blot*), compared to non-transfected cells (Figure 2B, *DMSO blot*). This suggests that HDAC6-mediated p62 autophagic clearance seems to be impaired by Nef-mediated HDAC6 degradation, and this Nef activity is, in turn, not blocked by 3-MA (Figure 2A, *3-MA blot*). Moreover, in the presence of an inhibitor of vacuolar H+-ATPases (V-ATPases), Bafilomycin A1, which abrogates endosome-lysosome acidification (Werner et al., 1984; Bowman et al., 1988; Yoshimori et al., 1991; Paroutis et al., 2004), Nef does not promote HDAC6 degradation (Figure 2B, *Bafilomycin A1 blots*). Hence, the impairment of Nef-mediated HDAC6 degradation by this inhibitor could be indicative of an HDAC6-suffered endocytic/lysosomal degradation pathway triggered by Nef.

To analyze the effects on Nef-induced HDAC6 degradation, we next assayed a combination of two proteinase inhibitors, as the broad-spectrum inhibitors of lysosomal cathepsins E-64d and the Pepstatin A, a cysteine and an aspartyl proteinase inhibitor, respectively (McGrath, 1999; Zhang et al., 2000; Muller et al., 2012; Verma et al., 2016; Agbowuro et al., 2018). We observed that a combination of these two inhibitors abrogated Nef-induced degradation of HDAC6 (Figure 2B, *E-64d* + *PepsA blot*), compared to mock cells (Figure 2B, *DMSO blot*). As shown in Figure 2B (*E-64d* + *PepsA blot*), the level of p62 or acetylated α-tubulin was similar to those observed under Bafilomycin A1 treatment (*Bafilomycin A1 blots*). The clearance of p62 and the low amount of acetylated α-tubulin detected are indicative of a functional HDAC6 enzyme, under these experimental conditions. Therefore, it is plausible to suggest that Nef does not affect HDAC6 enzymatic activity. Furthermore, the effect of the E-64d and Pepstatin A combination was compared to that of Bafilomycin A1 on the inhibition of Nef-mediated HDAC6 degradation (Figure 2B). Altogether these data prompted us to suggest that Nef could mediate its degradative action on HDAC6 by targeting this deacetylase enzyme at low pH organelles, where it would be degraded by the action of proteinases sensitive to Bafilomycin A1 and/or E-64d + Pepstatin A inhibitors, such as the endosomal and lysosomal cathepsins (Muller et al., 2012).

### Nef Mutant Lacking Endocytic Function Does Not Promote HDAC6 Degradation

**FIGURE 3:**
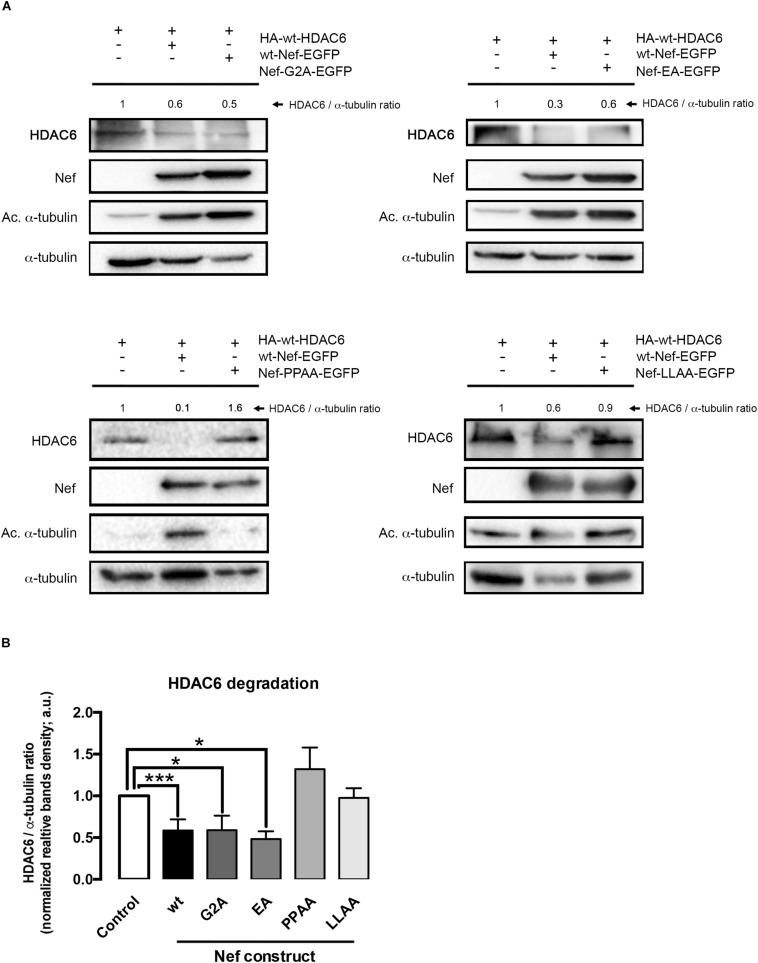
Characterization of different Nef mutants in their ability to promote HDAC6 degradation. **(A)** Quantitative western blot analysis of the effect of different Nef mutants on HDAC6 degradation, and compared to wt-Nef-triggered HDAC6 degradation. All experiments were performed in HEK-293T cells, transfected with 1 μg of HA-wt-HDAC6 and 0.5 μg of each assayed C-terminal EGFP-tagged Nef mutant: Nef-G2A, Nef-EA, Nef-PPAA, and Nef-LLAA. HDAC6/α-tubulin ratios are shown in arbitrary light units (a.u.). In all western blots, α-tubulin is the control for total protein. **(B)** Histograms show quantification of HDAC6/α-tubulin western blot intensity band ratios, under any experimental condition, indicating that Nef-PPAA and Nef-LLAA are not able to degrade HDAC6. Data are expressed as mean ± S.E.M. of three independent experiments. ^∗∗∗^*P* < 0.001 and ^∗^*P* < 0.05 values for Student’s *t-*test, respectively.

Considering that the anti-HIV-1 enzyme HDAC6 (Valenzuela-Fernandez et al., 2005, 2008; Valera et al., 2015; Casado et al., 2018; Cabrera-Rodriguez et al., 2019) is a non-cell-surface protein, mainly expressed at cytoplasm (Hubbert et al., 2002; Valenzuela-Fernandez et al., 2005, 2008; Valera et al., 2015), we first studied the degradation of HDAC6 in cells expressing a non-myristolated Nef mutant, Nef-G2A (Figure 3), which is unable to directly anchor to membranes (Saksela et al., 1995; Lee et al., 1996; Piguet et al., 1998; Mandic et al., 2001; Fackler and Baur, 2002; Madrid et al., 2005; Chaudhuri et al., 2007; Arhel and Kirchhoff, 2009; Kwak et al., 2010; Nobile et al., 2010; Foster et al., 2011). We observed that Nef-G2A-EGFP promotes HDAC6 degradation to a similar extent that wt-Nef-EGFP does (Figure 3A, quantified in Figure 3B). This finding suggests that Nef is able to target cytoplasmic HDAC6. We next assayed two Nef-defective mutants, Nef-EA and Nef-LL/AA, for its association with the adaptor proteins AP-1, -2, and -3, host proteins governing internalization, recycling, and lysosomal degradation processes. We observed that Nef-EA is able to promote HDAC6 degradation (Figure 3A, *quantified in* Figure 3B), whereas the Nef-LLAA mutant loses this degradative ability (Figure 3A, *quantified in* Figure 3B). Thus, this di-Leucin motif (L164-L165) in Nef appears to be critical for its degradative action on HDAC6. On the contrary, the Nef-EA mutant, reported to conserve the ability to interact with AP-1 (Bresnahan et al., 1998), promotes HDAC6 degradation (Figure 3A, *quantified in* Figure 3B). Taken into account our results using chemical inhibitors and Nef-mutants, it is conceivable that Nef interacts with and drives intracellular HDAC6 degradation into acidic, endocytic/lysosomal compartments related to the Nef/AP-1 trafficking route.

### Nef Mutant Lacking the Proline-Rich SH3-Ligand Domain Does Not Promote HDAC6 Degradation

Another key motif in Nef is a proline rich sequence (P69xxP72xxP75xR), a SH2/3-binding domain with effects on cell signaling. This Nef motif has been reported to interact with the p59Hck kinase and to be key for viral replication and egress (Saksela et al., 1995). We next assayed the Nef-P72xxP75/AxxA (Nef-PPAA) mutant, impaired for its interaction with SH2/3 domains of the Src-family of tyrosine kinases and dispensable for CD4 internalization in non-T cells (Saksela et al., 1995). We observed that Nef-PPAA did not promote HDAC6 degradation (Figure 3A, *quantified in* Figure 3B). This fact may indicate that this motif is involved in Nef-mediated HDAC6 interaction and/or processing, or that a conformational change in the mutated viral protein abrogates the degradative activity observed with the wt-Nef (Figures 1–3). This possibility is further analyzed in this work.

### Nef Co-immunoprecipitates and Co-distributes With HDAC6

**FIGURE 4:**
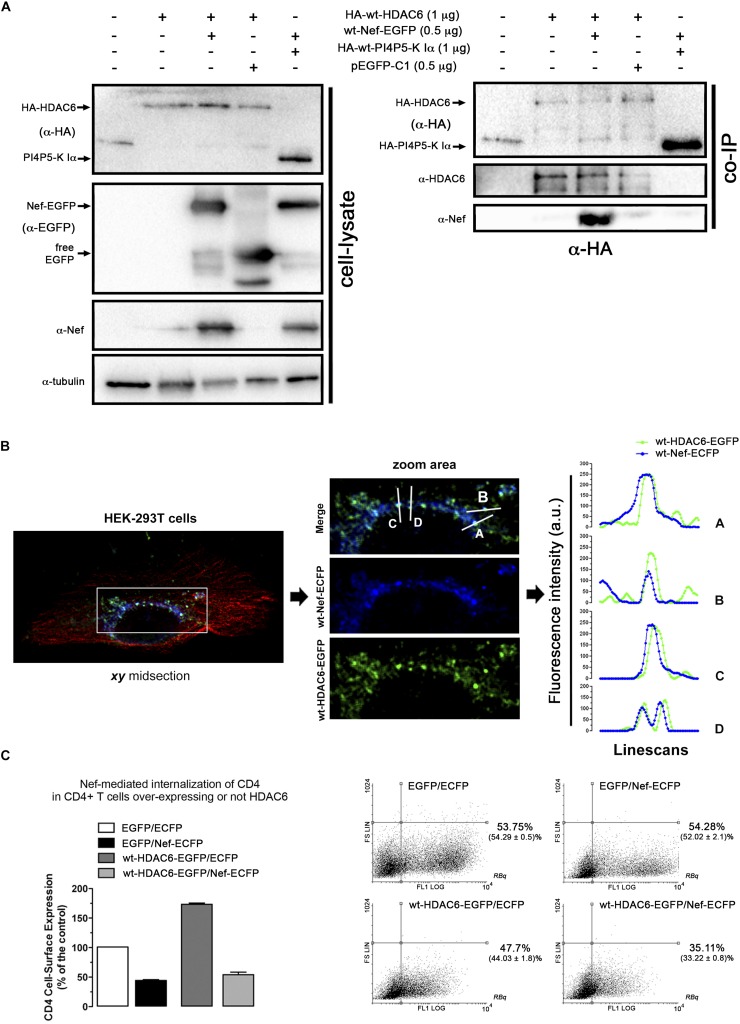
Nef co-immunoprecipitates and co-distributes with HDAC6. **(A)** Biochemical immunoprecipitation analysis of the HDAC6/Nef interaction in permissive HEK-293T cells. *Left panel*; western blot analysis of the input expression of the different over-expressed proteins, in cells expressing HA-wt-HDAC6, or wt-Nef-EGFP, or both HA-wt-HDAC6/wt-Nef-EGFP constructs, HA-wt-PI4P5-K Iα or pEGFP-C1 plasmid for free EGFP protein (these last two constructs are for tag and specificity of binding controls). Cell lysates under control conditions are indicated by the entire (-) lane which represents cells transduced with an empty pcDNA3.1 vector. *Right panel*; these cell lysates were further subjected to co-immunoprecipitation (co-IP) with anti-HA Ab, followed by immunoblotting with anti-HDAC6, anti-Nef and anti-HA specific Abs. Data are from a representative experiment of three. **(B)** Fluorescent confocal merge image, *xy* midsection, shows HDAC6 (green) and Nef (blue) co-distribution, together with microtubules (red) in HEK-293T cells. In the associated zoom area is shown the expression and co-distribution (merge) of these Nef/HDAC6 proteins. Line scans analysis show quantification of the fluorescence intensity profiles (in arbitrary light units, a.u.), associated to these two proteins, and representing the amount of these proteins and their co-localization along each differently oriented line scans (A-D). Data are from a representative experiment of three. **(C)** Flow cytometry analysis of Nef-mediated down-regulation of CD4 in permissive CEM.NKR-CCR5+/CD4+ T-cells (EGFP/Nef-ECFP), or in cells over-expressing HDAC6 and Nef (wt-HDAC6-EGFP/Nef-ECFP). CD4 cell-surface expression in control, non-Nef-, non-HDAC6-treated cells (EGFP/ECFP) and in cells only over-expressing HDAC6 are also shown (wt-HDAC6-EGFP/ECFP). Data are mean ± S.E.M. of three independent experiments. Right Dot-Plots show a representative flow cytometry analysis for the number of cells transiently expressing the different constructs. Numbers indicate the percent of cells positive for fluorescence protein expression (FL1 channel) in the Right Bottom quadrant (*RBq*). In parentheses, data are mean ± S.E.M. of three independent experiments.

We next sought to study whether Nef interacts with HDAC6. Co-immunoprecipitation experiments (co-IP) were performed in HEK-293T cells transiently expressing Nef-EGFP and HA-wt-HDAC6. We observed that HA-wt-HDAC6 specifically co-immunoprecipitates with Nef-EGFP (Figure 4A, *a-HA co-IP*). As a control for co-IP specificity, we expressed HA-wt-PI4P5-K Iα, a kinase involved in efficient HIV-1 viral entry and infection (Barrero-Villar et al., 2008). Data obtained indicate that Nef-EGFP did not interact with the HA-wt-PI4P5-K Iα kinase (Figure 4A, *a-HA co-IP*). Altogether these data confirm that Nef and HDAC6 co-immunoprecipitate together, maybe through a direct interaction or being part of an associated complex of proteins. Next, using fluorescence confocal microscopy, we observed that transiently over-expressed Nef-ECFP and HDAC6-EGFP co-distributed in permissive HEK-293T cells (Figure 4B, *merged image and line scans in the zoom area*). Moreover, Nef-ECFP activity on CD4 expression was assayed by flow cytometry, in permissive CEM.NKR-CCR5 CD4+ T-cells (Figure 4C). As expected, over-expressed Nef efficiently decreases cell-surface levels of CD4. However, the over-expression of wt-HDAC6-EGFP appears to enhance the amount of cell-surface CD4, which is similarly down-regulated by Nef (Figure 4C). Altogether these data suggest that functional Nef co-distributes and co-immunoprecipitates with HDAC6, and maintains the ability to internalize CD4 at the same time, even in cells over-expressing HDAC6.

**FIGURE 5:**
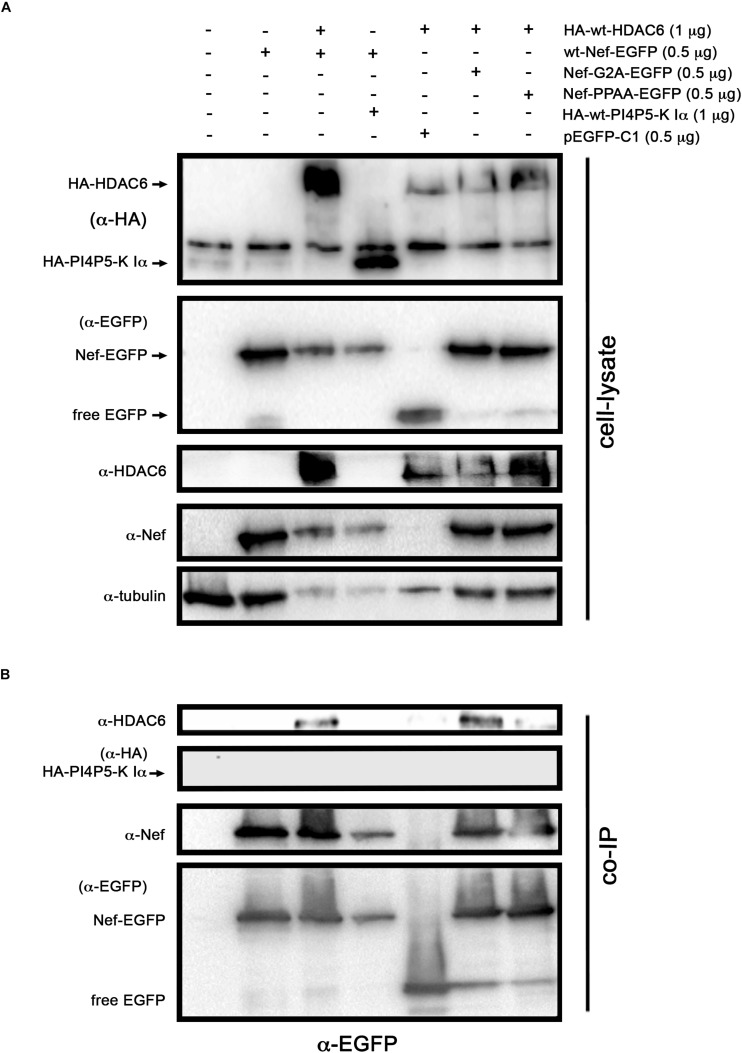
Study of the ability of two different Nef mutants, presenting divergence to target HDAC6, to co-immunoprecipitate with HDAC6. Biochemical immunoprecipitation analysis of the interaction of Nef-G2A and Nef-PPAA Nef mutants with HDAC6 in HEK-293T cells. **(A)** Western blot analysis of the input expression of the different over-expressed C-terminal EGFP-tagged Nef constructs (wt or mutants: Nef-G2A or Nef-PPAA), cells expressing HA-wt-HDAC6 or HA-wt-PI4P5-K Iα (these two constructs are for tag and specificity of binding controls), free EGFP (pEGFP-C1 expressing cells for EGFP control) or co-expressing HA-wt-HDAC6 with wt-Nef-EGFP, Nef-G2A-EGFP, or Nef-PPAA-EGFP constructs. Cell lysates under control conditions, are indicated by the entire first left column which represents cells transduced with an empty pcDNA3.1 vector. **(B)** These cell lysates were further subjected to co-immunoprecipitation (co-IP) with anti-EGFP Ab, followed by immunoblotting with anti-HDAC6, anti-HA, anti-Nef and anti-EGFP specific Abs. Data are from a representative experiment of three.

To further explore the potential regions on Nef involved in the interaction with HDAC6, we assayed a co-IP experiment in cells over-expressing the Nef-G2A or Nef-PPAA mutant together with HDAC6 (Figure 5A). We observed that wt-Nef and Nef-G2A specifically co-immunoprecipitate with HA-wt-HDAC6, while the Nef-PPAA mutant showed a reduced interaction with the deacetylase (Figure 5B, *α-EGFP co-IP*). As controls, we observed that HA-wt-HDAC6 did not co-immunoprecipitate with free EGFP, and wt-Nef is not pulling-down PI4P5-K Iα (Figure 5B, *α-EGFP co-IP*).

Therefore, these data suggest that Nef co-distributes and co-immunoprecipitates with and promotes HDAC6 degradation. This Nef activity seems not to require its expression in membranes, since Nef-G2A mutant is fully active. Moreover, results indicate that the integrity of the P72xxP75 motif is required for the association with HDAC6, either directly or indirectly. Indeed, Nef-PPAA mutant does not efficiently co-immunoprecipitates with HDAC6, which correlates with its poor activity to promote HDAC6 degradation. Our results suggest that the P72xxP75 motif is central for Nef activity in the targeting of HDAC6, in order to exert its proviral function.

### HDAC6 Is a Restriction Factor for HIV-1 Counteracted by Nef

**FIGURE 6:**
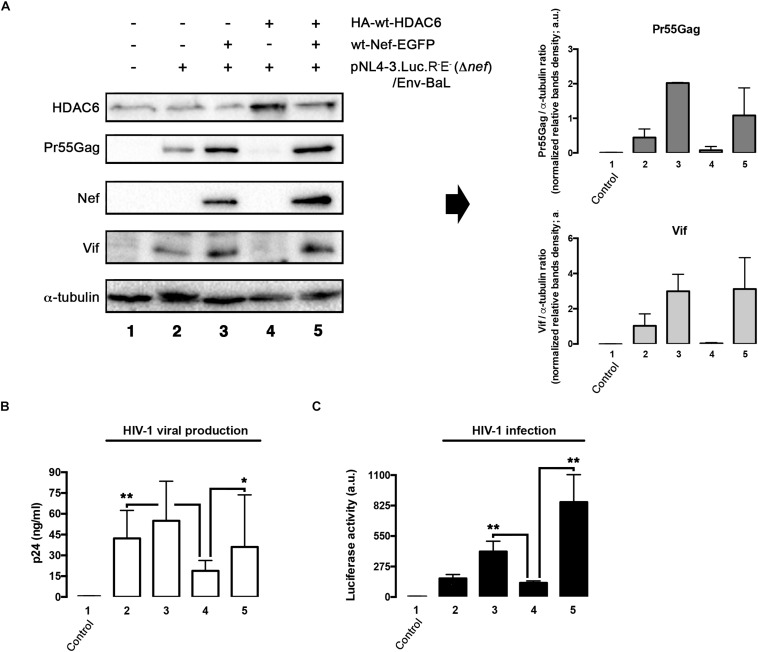
HDAC6 degrades Pr55Gag and Vif, inhibiting viral production and virus infection. Whereas Nef counteracts HDAC6, assuring Pr55Gag and Vif stability, and subsequent viral production and virus infection. **(A)** Quantitative western blot analysis of proviral pNL4.3-Luc-R-E-(Δ*nef*)-derived Pr55Gag and Vif proteins, in HIV-1(bering R5-tropic Env-BaL) virions-producing HEK-293T cells, over-expressing either HDAC6 (lane 4), Nef (lane 3) or both HDAC6/Nef constructs (lane 5). Lane 1 represents control, untrasduced cells that do not produce virions, and lane 2 pNL4.3.Luc.R-E-(Δ*nef*)/Env-BaL virus packaging cells. Right Histograms quantify the amounts of Pr55Gag and Vif viral proteins, normalized by total α-tubulin, under any experimental condition. Data are mean ± S.E.M. of three independent experiments. **(B)** Quantitative analysis of viral production in supernatants of HEK-293T cells, measured by quantitative p24-ELISA test, under any experimental condition as indicated in **(A)**. **(C)** Effects of HDAC6 and Nef-mediated HDAC6 targeting on HIV-1 infection assayed in CEM.NKR.CCR5 permissive T-cells, incubated with synchronous HIV-1 (Δ*nef*)/Env-BaL viral inputs, of virus isolated from supernatants of Nef construct-expressing cells of **(A)**. Data are mean ± S.E.M. of three independent experiments. ^∗∗^*P* < 0.01 and ^∗^*P* < 0.05 values Student’s *t*-test, respectively.

**FIGURE 7:**
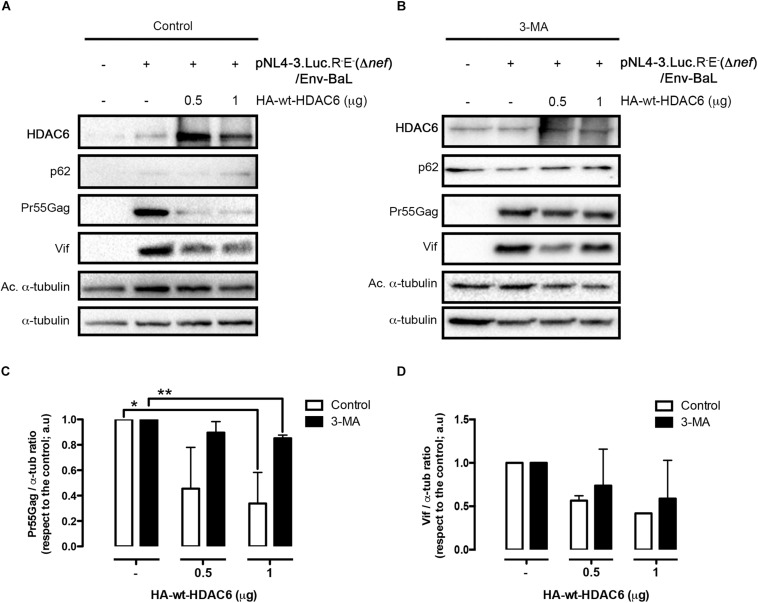
HDAC6-mediated Pr55Gag and Vif degradation is inhibited by 3-MA. Quantitative western blot analysis of dose-response HDAC6-mediated viral Pr55Gag and Vif degradation in virus-packaging HEK-293T cells, under control (vehicle PBS) **(A)** and 3-MA **(B)** conditions. When indicated, HEK-293T cells were transfected with proviral pNL4.3.LucR-E-(Δ*nef*) (1 μg) and BaL.01 *env* (1 μg) plasmids, and HA-wt-HDAC6. **(C,D)** Show histogram quantifications of the inhibitory effect of 3-MA on HDAC6-mediated Pr55Gag and Vif autophagic degradation, respectively. Data are mean ± S.E.M. of three independent experiments carried out in quadrupled: ^∗∗^*P* < 0.01 and ^∗^*P* < 0.05 values Student’s *t*-test, respectively.

We next aimed to understand the importance of Nef-mediated HDAC6 degradation in the late steps of the HIV-1 viral cycle. In HEK-293T cells, producing HIV-1 Δ*nef* viral particles, we observed that over-expression of HDAC6 efficiently promotes Pr55Gag degradation (Figure 6A, *lane 4; quantified in right histogram*), thereby inhibiting viral production (Figure 6B, *lane 4*). Moreover, we observed that HDAC6 concomitantly degrades Vif, under this HIV-1 Δ*nef* experimental condition (Figure 6A, *lane 4; quantified in right histogram*). The anti-Vif activity is mediated by the HDAC6-proautophagy function, thus impairing HIV-1 infectiveness as we reported (Valera et al., 2015), and correlates with a less infectious cell-supernatant, as further measured in early infection experiments in permissive T-cells (Figure 6C, *lane 4*). Remarkably, over-expression of functional Nef degrades HDAC6, either the endogenous or the over-expressed enzyme (Figure 6A, *lanes 3 and 5*, respectively). Under these experimental conditions, Pr55Gag and Vif are stabilized (Figure 6A, *lanes 3 and 5, quantified in right histograms*), observing an enhancement in viral production (Figure 6B, *lanes 3 and 5*), and supernatant infectivity as measured in permissive T-cells (Figure 6C, *lanes 3 and 5*), compared to cells only over-expressing HDAC6 (Figure 6, *lane- and histograms-4*). This proviral function is strictly dependent on Nef expression and HDAC6 targeting, as it was not observed in *nef*-defective viruses. Hence, Pr55Gag and Vif expression levels were significantly reduced in cells producing HIV-1 Δ*nef* virions, compared to those found in cells co-transduced with a functional *nef* construct (Figure 6A, *lanes 2, 3, and 5, quantified in right histograms*). Of note, the ability of wt-Nef to rescue Pr55Gag and Vif levels was, via degrading the endogenous levels of HDAC6, an almost 5 and 3-fold increase, respectively, compared with control condition, where Nef was absent. This competence was also reproduced in the presence of exogenously expressed HDAC6, where both viral proteins were recovered nearly completely. Also, in terms of viral production and infectivity, the impact of Nef always yields a gain of fitness with or without the overexpression of HDAC6 (until double production of viral particles, and as far as 6-fold increase in the capacity to infect). These results indicate that endogenous HDAC6 is able to restrict viral production and infection, in the absence of Nef. Indeed, over-expressed HDAC6 promotes Pr55Gag and Vif degradation in a dose-dependent manner (Figure 7A, and quantified in Figures 7C,D, respectively). This degradative process appears to occur by autophagy, as monitored by concomitant p62 clearance (Figure 7A), and by the inhibitory action of 3-MA which blocks HDAC6-mediated Pr55Gag and Vif degradation, thus stabilizing p62 (Figure 7B, and quantified in Figures 7C,D).

**FIGURE 8:**
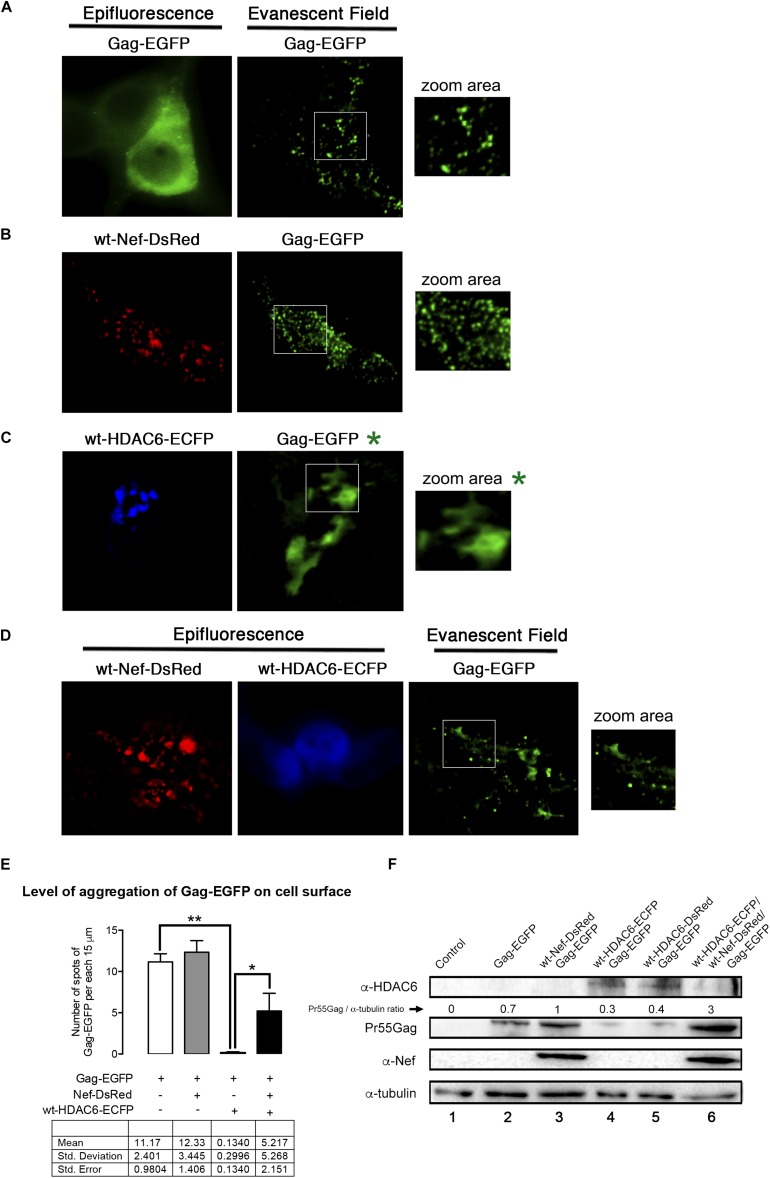
Nef assures Gag localization and aggregation at plasma membrane by targeting HDAC6 (TIRFM study). A series of epifluorescence and TIRFM images, in HEK-293T living cells, showing the expression, localization and aggregation pattern of the Gag-EGFP protein at plasma membrane, monitored by the EGFP associated fluorescence observed in the evanescent field, under different experimental conditions. **(A)** Images correspond to data obtained in control, Gag-EGFP expressing cells. Zoom area image shows Gag-EGFP aggregates at plasma membrane. **(B)** Images correspond to data obtained in wt-Nef-DsRed and Gag-EGFP co-expressing cells. Zoom area image shows Nef enhanced Gag-EGFP aggregates at plasma membrane. **(C)** Images correspond to data obtained in wt-HDAC6-ECFP and Gag-EGFP co-expressing cells. Zoom area image shows HDAC6-mediated impairment of Gag-EGFP aggregates at plasma membrane. In fact, under the evanescent field is not possible to observe Gag-EGFP aggregates, under this experimental condition. We need to explored inside the cell to detect EGFP-associated fluorescent without any specific pattern. The associated, non-evanescent field images are indicated by a green asterisk. **(D)** Images correspond to data obtained in wt-Nef-DsRed, wt-HDAC6-ECFP and Gag-EGFP co-expressing cells. Zoom area image, under the evanescent field, shows Nef-mediated enhancement of Gag-EGFP aggregates at plasma membrane, by Nef-targeting of HDAC6 which is weak observed by epifluorescence. Data are representative of three independent experiments. **(E)** Histograms show quantification of the aggregation level of Gag at plasma membrane, monitored in the evanescent field, under the experimental conditions indicated in **(A–D)**. To quantify the degree of Gag-EGFP aggregation at plasma membrane, TIRFM images were background subtracted using MetaMorph and analyzed plotting 3 lines of 15 μm-length along the cell diameter. These data and quantitative analysis is presented in the table underneath. The difference observed in the Gag-EGFP punctuated pattern between the control condition (Gag-EGFP) and cells coexpressing Gag-EGFP and wt-HDAC6-ECFP is significant ^∗∗^*P* = 0.0078 value of Student’s *t*-test. HDAC6 inhibits Gag location and aggregation at plasma membrane by 98% respect to control condition **(A)**. The recovery of the punctate expression pattern over-expressing Nef was also significant ^∗^*P* = 0.0277 value of Student’s *t-*test. Signal was scored positive when the fluorescence of the spots in the line scans were at least mean ± 2SD of the local background. Data were pooled in histograms that show averaged number of aggregates per cell. Data are mean ± S.E.M. of six independent experiments. **(F)** Quantitative western blot analysis of the different proteins expressed in cells used in TIRFM studies from **(A–D)**. We observed that fluorescent tags do not affect the ability of HDAC6 (either C-terminal ECFP or DsRed tagged) to degrade Gag-EGFP, the ability of Nef (C-terminal DsRed tagged) to target HDAC6 and to stabilize and enhance Gag protein level of expression. Pr55Gag-EGFP/α-tubulin rations are shown. α-tubulin bands represent the control for the total amount of protein. A representative western blot is shown of the six independent experiments performed, corresponding with cells assayed in TIRFM **(A–D)** panels.

We next aimed to study, by total internal reflection fluorescence microscopy (TIRFM), the ability of HDAC6 to alter Pr55Gag location and aggregation pattern at plasma membrane of living cells, and the effect exerted on them by Nef (Figure 8). As reported, recombinant Gag-EGFP directs the budding of viral-like particles (VLPs) from cells (Pornillos et al., 2003; Muller et al., 2004; Garcia-Exposito et al., 2011). We therefore used a Pr55Gag-EGFP construct (indicated as Gag-EGFP) that allows the monitoring and quantification of Pr55Gag aggregation at plasma membrane in single cell by tracking the associated fluorescence in the evanescent field (Barrero-Villar et al., 2009; Barroso-Gonzalez et al., 2009a, b; Garcia-Exposito et al., 2011). Over-expressed Gag-EGFP presents a homogeneous aggregation pattern at plasma membrane, as observed under the evanescent field in HEK-293T cells (Figure 8A, *evanescent field image*; and quantified in Figure 8E). Over-expression of a wt-Nef-DsRed construct, which stabilizes Gag-EGFP (Figure 8F, *track 3, and compared to track 2*), leads to an increase in the number of Gag-EGFP aggregates at plasma membrane (Figure 8B, *evanescent field image*; and quantified in Figure 8E). Here, we observed again that Nef counteracts the antiviral effect of the endogenous HDAC6, thus favoring Gag-EGFP location and aggregation at plasma membrane (Figure 8B, *evanescent field image*; and quantified in Figure 8E). Furthermore, HDAC6 over-expression (Figure 8C, *wt-HDAC6-ECFP epifluorescence image*) abrogates Gag-EGFP aggregation at plasma membrane. In fact, we do not observe any Gag-associated aggregates under the evanescent field (quantified in Figure 8E). EGFP-associated fluorescent is only observed when we modify the angle of the laser bin away from the angle required to create an evanescent field. This means that we are far from the plasma membrane, inside the cell, and that we are not able to detect any aggregation pattern for Gag. Residual fluorescence signal detected by epifluorescence is likely related to free EGFP (Figure 8C, *Gag-EGFP^∗^ and associated zoom area^∗^*). In this matter, when lysates of these cells are biochemically analyzed, Pr55Gag-EGFP is not detected under this experimental condition (Figure 8F, *track 4*). The expression of HDAC6-ECFP abolished the Gag-EGFP aggregation in more than 98% of cases, but the only expression of wt-Nef-DsRed could recover almost half compared with the control conditions (Figure 8E). Hence, wt-HDAC6-DsRed degrades Gag-EGFP (Figure 8F, *track 5*), indicating that the C-terminal fluorescent tag is neither affecting nor responsible for the HDAC6 degradative action. Remarkably, wt-Nef-DsRed over-expression (Figure 8D, *associated epifluorescence image*) restores Gag-EGFP export to, and aggregation at, plasma membrane (Figure 8D, *evanescent field image*; and quantified in Figure 8E), correlating with the degradation of wt-HDAC6-ECFP and the concomitant Pr55Gag (Gag-EGFP) stabilization observed in the associated cell lysates (Figure 8F, *track 6*). Therefore, HDAC6 degrades Pr55Gag inhibiting its localization and aggregation pattern at plasma membrane. Nef targets HDAC6 neutralizing its anti-HIV-1 action, in order to assure Pr55Gag stability, and its location and aggregation pattern at plasma membrane.

Taking together all the data, it is plausible to propose that HDAC6 is an anti-HIV-1 restriction factor, limiting viral production and infection, acting in the late steps of the viral cycle. Indeed, Nef targets HDAC6, being key to guarantee these late steps of the viral cycle.

### Full-Length Nef Targets HDAC6 Assuring Viral Production and Infection

**FIGURE 9:**
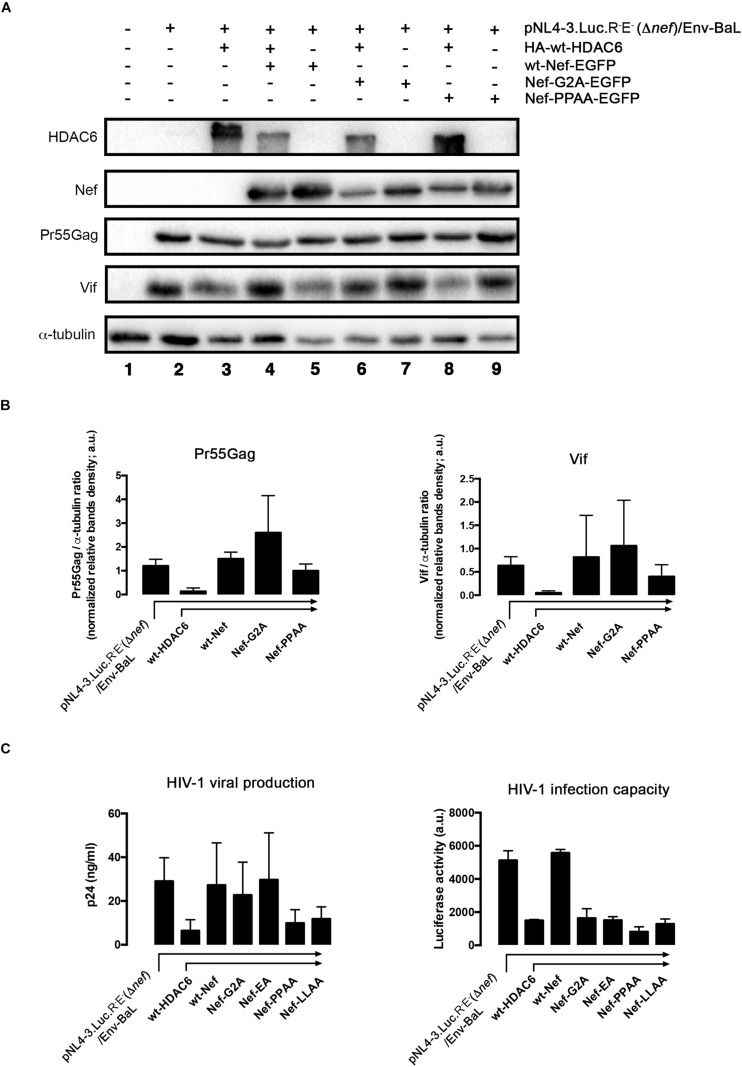
Only full-length functional Nef neutralizes HDAC6 to assure virus production and infection capacities. **(A)** Quantitative western blot analysis of proviral pNL4.3-Luc-R-E-(Δ*nef*)-associated Pr55Gag and Vif proteins expression, in HIV-1 (R5-tropic Env-BaL) virus packaging HEK-293T cells (from lanes 2 to 9), after over-expressing HDAC6 (lane 3), Nef (lane 5) or both HDAC6/Nef constructs conditions (lane 4). The effect of Nef-G2A and Nef-PPAA mutants, expressed alone (lanes 7 and 9, respectively) or together with HDAC6 (lanes 6 and 8, respectively) on Pr55Gag and Vif stability are similarly shown. Lane 1 represents control for non-transduced, non-virus producing cells, and lane 2 is the control for HIV-1 (Δ*nef*/*env*-BaL) virus production. A representative experiment of three is shown. **(B)** Histograms quantify the intensities of western blot bands, representing the amounts of Pr55Gag and Vif viral proteins detected, under any experimental condition from **(A)** experiments, and normalized by total α-tubulin. Data are mean ± S.E.M. of three independent experiments. **(C)** *Left*; Quantitative analysis of the effect of different Nef constructs on viral production in supernatants of over-expressing-HDAC6, virus-packaging HEK-293T cells, measured by a quantitative p24-ELISA test, under similar experimental conditions as in **(A)**. *Right*; Effects of different Nef constructs on HIV-1 infection capacity. Synchronous HIV-1 (Δ*nef*/*env*-BaL) viral inputs, from supernatants of *left*-panel **(C)** experimental conditions, were incubated with CEM.NKR.CCR5 permissive T-cells, and assayed in luciferase-quantified viral infection experiments. Data are mean ± S.E.M. of three independent experiments. In **(B,C)**, a.u., arbitrary light units.

To further decipher which Nef determinants are responsible for HDAC6 neutralization, we studied the effects of different mutants on viral production. We observe that wt-Nef and the Nef-G2A, but not the Nef-PPAA mutant, efficiently abrogate HDAC6-mediated Pr55Gag and Vif degradation (Figure 9A; quantified in Figure 9B). We also observed that HDAC6-mediated inhibition of virus production was neutralized by wt-Nef, Nef-G2A, and Nef-EA mutants (Figure 9C, *viral production histograms*). In contrast, Nef-PPAA and Nef-LLAA mutants cannot restore efficient viral production (Figure 9C, *viral production histograms*), in agreement with their inability to degrade HDAC6 (Figure 3 and Figure 9A).

**FIGURE 10:**
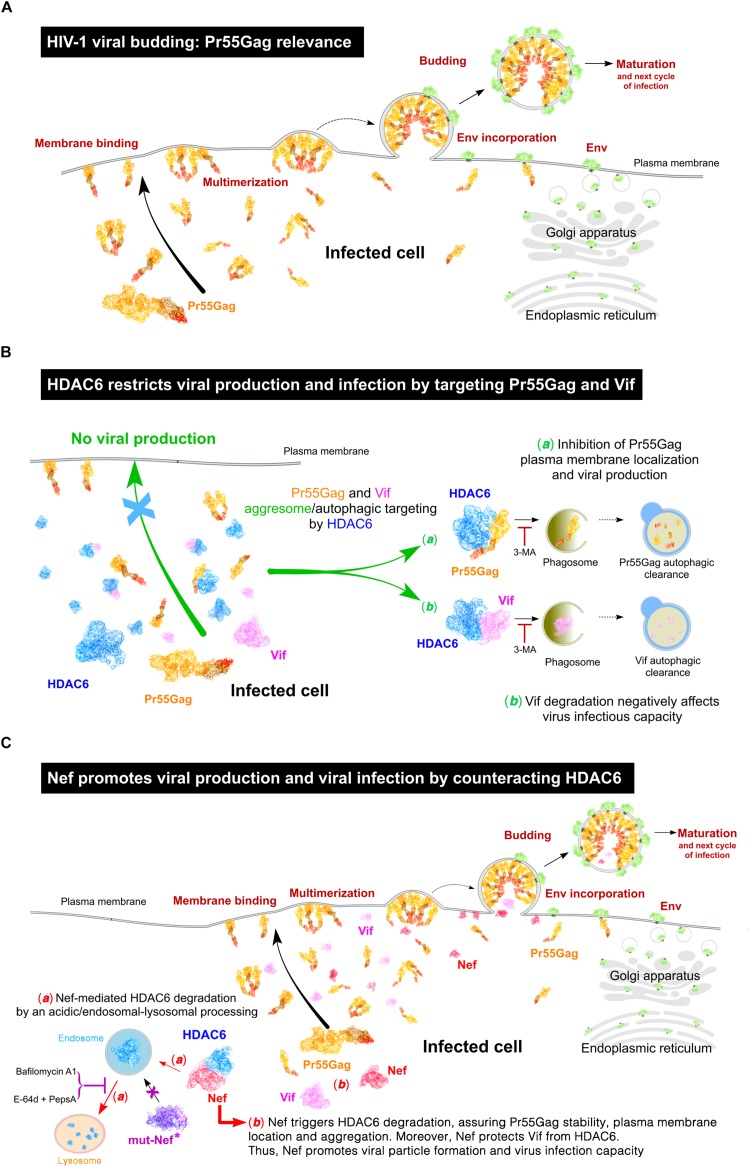
Schematic representation of the interplay between HDAC6 and HIV-1 viral proteins Nef, Pr55Gag and Vif, in the control and fostering of viral production and virus infection. **(A)** Schematic illustration showing the different stages of HIV-1 assembly, budding and maturation, all processes mainly driven by the HIV-1 structural Gag protein (Pr55Gag). **(B)** Schematic illustration of the anti-HIV-1 action of HDAC6 inhibiting viral production and virus infection capacity, thereby targeting Pr55Gag and Vif to the aggresome/autophagic clearance pathway. This process is blocked by 3-methyladenine (3-MA), a chemical inhibitor of aggresome/phagosome formation. **(C)** Schematic illustration of the HIV-1 Nef proviral action counteracting HDAC6. Nef targets HDAC6 to degradation by an acidic-endocytic/lysosomal route, where endosomal and lysosomal cathepsins proteinases could be involved, which are sensitive to E-64d and Pestatin A proteinase inhibitors, and Bafilomycin A1, an inhibitor of vacuolar H+-ATPases (V-ATPases) that abrogates endosome-lysosome acidification. Indeed, the Nef mutant (mut-Nef^∗^) Nef-LLAA, disconnected of the AP-1 endocytic route, cannot trigger HDAC6 degradation. Similarly, Nef-PPAA mut-Nef^∗^, which cannot co-immunoprecipotate with and degrade HDAC6, is unable to restore efficient viral production. The antiviral HDAC6 functions are only neutralized by functional full-length Nef and Nef mutants able to target HDAC6, such Nef-G2A and Nef-EA. This new Nef proviral function assures Pr55Gag and Vif stabilization, allowing Pr55Gag localization and aggregation at plasma membrane, viral egress and efficient virus infection capacity. HDAC6 and HIV-1 Pr55Gag, Nef and Vif proteins are represented in these illustrations by using their related molecular structures (http://www.rcsb.org/structure/3PHD and http://cdn.rcsb.org/pdb101/learn/resources/structural-biology-of-hiv/index.html, respectively) hold by the Protein Data Bank archive (^©^ RCSB PDB; Berman et al., 2000). Scheme illustrations created by David Reyes (@SciArt3D FabLab-ULL).

Next, we analyzed the infection capacity of virions produced under these experimental conditions. In permissive CEM.NKR-CCR5 T-cells, we assayed virions obtained in packaging cells expressing different Nef constructs and the same pNL4.3-Δ*nef* backbone (see section Materials and Methods). We observed that Δ*nef*-virions lose their infection capacity when viral particles are produced in the presence of over-expressed HDAC6 (Figure 9C, *HIV-1 infection capacity/HDAC6 histogram*). As expected, infectivity levels were restored in the presence of full-length Nef (Figure 9C, *HIV-1 infection capacity/wt-Nef histogram*). Despite its ability to target HDAC6, stabilizing Pr55Gag, virions obtained with the Nef-G2A mutant are poorly infectious (Figure 9C, *HIV-1 infection capacity/Nef-G2A histogram*). Interestingly, the small amounts of viral particles produced in the presence of wt-HDAC6 and the Nef-PPAA mutant, which is unable to target HDAC6 (Figures 3, 9), also presented lower infection capacities (Figure 9C, *HIV-1 infection capacity/Nef-PPAA histogram*). Similar results are obtained in infection experiments with virions obtained with the Nef-LLAA mutant (Figure 9C, *HIV-1 infection capacity/Nef-LLAA histogram*), which cannot target HDAC6. We and others have previously reported a weak infection capacity of HIV-1 obtained with Nef-LLAA (Madrid et al., 2005; Pizzato et al., 2007; Xu et al., 2009; Basmaciogullari and Pizzato, 2014). Interestingly, we observed that virions produced with the Nef-EA mutant, which is able to target HDAC6, present a weak infection capacity (Figure 9C, *HIV-1 infection capacity/Nef-EA histogram*). All these Nef mutants have already been reported to be responsible for a defect in virus infection capacity (reviewed in Vermeire et al., 2011), explaining our results. Therefore, all these data prompted us to suggest that HDAC6 targets Pr55Gag and Vif, limiting viral production and infection, antiviral functions that are only entirely neutralized by full-length functional Nef (see Figure 10, *summary illustrations*). Hence, HDAC6 appears to be an important anti-HIV-1 restriction factor.

## Discussion

In the present work, we propose a new function for Nef through targeting HDAC6, thereby neutralizing its antiviral functions. HDAC6 appears to limit viral production and infection by promoting the autophagic degradation of Pr55Gag and Vif. In this matter, Nef seems to promote HDAC6 degradation, in a dose-dependent manner. Nef co-distributes and co-immunoprecipitates with HDAC6, driving its clearance by an acidic, endosomal/lysosomal degradative pathway. This Nef-mediated anti-HDAC6 activity stabilizes Pr55Gag, assuring Pr55Gag location and aggregation at plasma membrane, as observed by TIRFM, and favoring efficient viral production. Moreover, and instead with HDAC6 over-expression conditions, nascent virions obtained in the presence of functional Nef present enhanced infection capacities compared to Δ*nef*-virions, which are poorly produced in the presence of HDAC6, as occurred with Nef mutants unable to target HDAC6. Moreover, Nef-mediated HDAC6 targeting restores Vif stability, which is crucial for viral infectiveness, as we reported (Valera et al., 2015).

Our data shows for the first time that HIV-1 Nef degrades HDAC6. This event leads to an increase in α-tubulin acetylation, a main HDAC6 substrate. This α-tubulin post-transductional modification is required for efficient HIV-1 viral infection and replication (Valenzuela-Fernandez et al., 2005, 2008; Casado et al., 2018; Cabrera-Rodriguez et al., 2019). In terms of the Nef-associated route to degrade HDAC6, our results indicate that neither MG132 nor 3-MA altered the ability of Nef to degrade HDAC6 (Figures 2A,B). These data suggest that Nef is not targeting HDAC6 to the proteasome, and that Nef is not recruiting HDAC6 to the aggresome/autophagy degradative pathway. However, a V-ATPases inhibitor, Bafilomycin A1, which abrogates endosome, lysosome and others vesicles/organelles acidification (Yoshimori et al., 1991), avoids Nef-mediated HDAC6 degradation (Figure 2B, *Bafilomycin A1 blots*). A comprehensive study points to the idea that Bafilomycin A1 does not affect autophagosome-lysosome fusion (Klionsky et al., 2008b). Hence, its main action would rely on the inhibition of lysosome acidification (Fass et al., 2006; Yamamoto et al., 1998). In this matter, it has been described that in the presence of Bafilomycin A1, a high autophagic flux into the lysosome is still maintained, even if an accumulation of early autophagic vacuoles is observed (Mousavi et al., 2001). This event could account for the lower amounts of p62 we detected in the presence of Bafilomycin A1, together with the fact that this inhibitor abrogates Nef-mediated HDAC6 degradation. Then, HDAC6/p62-mediated autophagic flux could be still functional, accounting for the p62 clearance observed during Bafilomycin A1 treatment. In fact, Bafilomycin A1 appears to block fusion between late endosomes and lysosomes (Mousavi et al., 2001), affecting intracellular protein trafficking toward endosomal or lysosome compartments (Clague et al., 1994; van Weert et al., 1995; van Deurs et al., 1996; Mousavi et al., 2001). It is therefore plausible that some markers for autophagic flux and degradation, such as p62, are not significantly accumulated by the general action of Bafilomycin A1 (Klionsky et al., 2008b), as we observed in this work. Hence, Bafilomycin A1 fails to elevate ubiquitinated proteins, as well as p62, unless the proteasome is affected (Myeku and Figueiredo-Pereira, 2011). We also observed that the combined action of E-64d and Pesptatin A inhibitors blocked the degradative Nef action on HDAC6. These two proteinases’ inhibitors act on low pH (McGrath, 1999; Zhang et al., 2000; Muller et al., 2012; Verma et al., 2016; Agbowuro et al., 2018), and are broad-spectrum inhibitors of lysosomal cathepsins, such as cysteine (E-64d) and aspartyl (Pepstatin A) proteinases (Muller et al., 2012). Altogether these data prompted us to suggest that Nef could mediate its degradative action on HDAC6 by targeting this anti-HIV-1 deacetylase enzyme at low pH organelles, where it would be degraded by the action of proteinases, such as endosomal and lysosomal cathepsins, sensitive to Bafilomycin A1 and/or E-64d + Pepstatin A inhibitors (Muller et al., 2012).

All these observations are consistent with data obtained with the Nef-LLAA mutant that lacks endocytic functions. The di-Leucin motif (L164-L165) in Nef appears to be critical for its degradative action on HDAC6, since this Nef mutant is unable to target HDAC6. Mutations in the di-Leucin motif have been reported to negatively affect Nef-mediated internalization and endosomal trafficking, such as for CD4 and other factors, through inhibiting Nef-mutant association with AP-1 (Bresnahan et al., 1998; Janvier et al., 2003a, b; Madrid et al., 2005). Interestingly, we observed that the Nef-EA mutant, reported to slightly interact with AP-1 and to internalize CD4 (Bresnahan et al., 1998), conserves the ability to trigger HDAC6 degradation. This Nef-EA mutant is altered in the Nef-ExxxLL160-165 sequence, critical for internalization, intracellular trafficking, and fade of several proteins and receptors targeted by HIV-1 Nef (Lee et al., 1996; Bresnahan et al., 1998; Janvier et al., 2003a, b; Madrid et al., 2005). Moreover, it seems that Nef promotes HDAC6 degradation without the need to be membrane-anchored for Nef, or to be a plasma membrane protein for HDAC6. Nef-G2A-mediated HDAC6 degradation and co-immunoprecipitation, observed in this work, point to this direction. Furthermore, results obtained with the Nef-PPAA mutant indicate that the P72xxP75 motif, in the PxxPxxPxR (69-77 aa) sequence of Nef, may be involved in the association with HDAC6, since Nef-PPAA mutant is not able to co-immunoprecipitate with HDAC6. Similarly, some Src-family tyrosine kinases that interact with Nef cannot be targeted by the Nef-PPAA mutant, as reported (Saksela et al., 1995; Lee et al., 1996; Arold et al., 1997; Manninen et al., 1998). In this matter, Nef-PPAA mutant also lack the capacity to promote HDAC6 degradation. This fact may be indicative that this motif is involved in Nef-mediated HDAC6 processing, or that a conformational change in the mutated Nef protein abrogates the activity presented by other parts of the molecule, as observed with the full-length Nef (Figures 1–3). Altogether, the data indicate that Nef interacts, directly or indirectly, with HDAC6, with the association of the integrity of the PPAA motif in Nef being important, and that mutants that co-immunoprecipitate with HDAC6 and conserve the endocytic functions of Nef are able to promote HDAC6 clearance through an acidic, endosomal/lysosomal pathway. Indeed, Nef promotes HDAC6 degradation without the need to be membrane-anchored for Nef or to be a plasma membrane protein for HDAC6, as results with the Nef-G2A mutant indicate.

Our data suggests that this Nef-mediated HDAC6 degradation is for the late steps of the HIV-1 viral cycle. In fact, HDAC6 represents a barrier for viral production and infection, as over-expression of HDAC6 promotes Pr55Gag and Vif degradation. This HDAC6 anti-Vif activity impairs HIV-1 infectiveness, as we reported (Valera et al., 2015). However, in the presence of functional Nef, HDAC6 cannot exert its anti-HIV-1 functions. Nef degrades HDAC6, stabilizing Pr55Gag and Vif, just assuring and enhancing viral production and infectiveness. TIRFM results help to monitor the effect exerted by HDAC6 on the Pr55Gag location and distribution pattern at plasma membrane, and how Nef neutralizes these effects. In the absence of over-expressed HDAC6, recombinant Gag presents a homogeneous aggregation pattern at plasma membrane, as observed under the evanescent field. Over-expression of Nef degrades endogenous HDAC6, stabilizes Gag protein, and enhances formation of Gag aggregates at plasma membrane. These data fit well with data obtained in cells producing viral particles, when Nef is over-expressed and neutralizes the basal anti-HIV-1 activity of the endogenous HDAC6. On the contrary, in the absence of Nef, HDAC6 over-expression targets Gag and avoids Gag location and aggregation at plasma membrane. Remarkably, Nef over-expression degrades over-expressed HDAC6 and restores Gag distribution and aggregation at plasma membrane. Therefore, HDAC6 degrades Pr55Gag, inhibiting its plasma membrane localization and aggregation pattern. Nef targets HDAC6 neutralizing its anti-HIV-1 action, in order to assure Pr55Gag stability, plasma membrane location and the aggregation pattern, thereby explaining the enhancement observed for viral egress. These observations were further confirmed by studying viral production and virus infection with different Nef mutants and over-expressing HDAC6. We observed that the antiviral HDAC6 functions were only neutralized by wt-Nef and Nef mutants able to target HDAC6, such Nef-G2A and Nef-EA, whereas mutants that cannot target HDAC6, such as Nef-PPAA and Nef-LLAA, are unable to restore efficient viral production. Hence, Δ*nef*-virions lose their infection capacity when viral particles are produced in the presence of over-expressed HDAC6. Indeed, this correlates well to the HDAC6-mediated Vif autophagy degradation that negatively affects HIV-1 infection, as reported (Valera et al., 2015). However, when viral particles are obtained in the presence of functional Nef, HDAC6 is degraded (either the endogenous or the over-expressed enzyme) and Vif is stabilized, assuring virion production with restored infection capacities.

Some interesting data were obtained when analyzing the infection capacity of viral particles obtained over-expressing HDAC6 together with the different Nef mutants. Despite stabilizing Pr55Gag, virions obtained with the Nef-G2A mutant were poorly infectious. This was previously reported for this mutant (Fackler et al., 2006), and may point to the necessity of Nef to be associated to the plasma membrane, in order to assure viral infectiveness by targeting other cell factors, during viral egress, such as dynamin 2, SERINC5, and 3 (Pizzato et al., 2007; Fackler, 2015; Rosa et al., 2015; Usami et al., 2015). In addition, viral particles produced under Nef-PPAA mutant condition also presented weak infection capacities. This observation is in accordance with a reported study suggesting that the Nef-SH3 binding motif, absent in Nef-PPAA, is required for enhanced growth of HIV-1 viruses, suggesting to the authors that the phenotypic effect of Nef may be explained by a higher infectivity of virus particles produced in Nef-expressing cells, rather a higher rate of virus production in infected cells (Saksela et al., 1995). Furthermore, P/A mutations in the Nef-P72xxP75 motif have been reported to entirely inhibit or impair Nef ability to enhance virion infectivity (Goldsmith et al., 1995; Pizzato et al., 2007; Foster et al., 2011). In this regard, our results suggest that Nef-PPAA poorly interacts with and does not target HDAC6, thereby being unable to prevent HDAC6 restrictions for viral production and infection, acting on Pr55Gag and Vif. Interestingly, we observed that virions produced with the Nef-EA mutant, despite being able to target HDAC6, present a low infection capacity. We have reported that Nef-induced disruption of the endocytic recycling compartment (ERC) is also lost with the Nef-EA(E160A) mutation (Madrid et al., 2005). It appears that the ^160^glutamate residue is important for canonical interactions of the di-Leucine motif, (E/D)XXXL(L/I) (Bonifacino and Traub, 2003). It is conceivable that despite its ability to target HDAC6 and assure viral production, and as observed with the Nef-LLAA mutant, Nef-EA may not be able to interplay with other LL (or LI) domains (reviewed in Foster et al., 2011), and to assure a post-viral egress function required for viral infectiveness. In this regard, virions obtained with the Nef-LLAA mutant present weak infection capacity, as we and others have previously reported (Madrid et al., 2005; Pizzato et al., 2007; Xu et al., 2009). This Nef mutant is unable to reduce the ERC compartment, composed of narrow diameter tubules derived from sorting endosomes (Gruenberg and Maxfield, 1995; Maxfield and McGraw, 2004), as Nef does (Madrid et al., 2005; Coleman et al., 2006; Pizzato et al., 2007; Lindwasser et al., 2008; daSilva et al., 2009; Laguette et al., 2009; Xu et al., 2009; Foster et al., 2011). It is conceivable that this Nef-LLAA mutant could also be unable to target or to recruit some cell factors, such as dynamin 2 (Pizzato et al., 2007), which is key for controlling viral infectiveness after viral particle formation. In fact, we observed that Nef-LLAA is unable to target HDAC6 and to neutralize HDAC6-mediated Pr55Gag degradation and inhibition of viral production and infection. This anti-HIV-1 activity of HDAC6 against Pr55Gag could be very important, and points to a similar action exerted by the cell-membrane metalloprotease TRAB domain-containing protein 2A (TRABD2A), which acts on resting CD4+ T-cells (Liang et al., 2019). Of note, HDAC6 also controls HIV-1 infectiveness by targeting Vif, as we observed and reported here (Valera et al., 2015). Therefore, only full-length functional Nef is able to target HDAC6 to restore viral production rates and viral infection capacities.

Altogether, these results prompted us to suggest that HDAC6 acts as a restriction factor, limiting viral production and infection by driving Pr55Gag and Vif viral proteins to degradation through an aggresome/autophagy route. Thus, for HIV-1, targeting HDAC6 appears to be critical for assuring viral production and virus infectivity, and that this could be a key proviral function of Nef. Hence, HDAC6 is counteracted by functional Nef which drives its clearance by an acidic, endocytic/lysosomal pathway. In this matter, Nef assures viral production and infection by targeting HDAC6, stabilizing Pr55Gag and Vif, thereby facilitating Pr55Gag location and aggregation at plasma membrane, and subsequent virus production and infection capacity (events summarized by schematic illustrations in Figure 10). Therefore, the interplay between Nef and HDAC6 may be key to the course of HIV infection and pathogenesis in infected individuals, and may contribute to develop new strategies against HIV.

## Data Availability Statement

All relevant datasets are contained within the manuscript.

## Author Contributions

SM-H and JE-H wrote the manuscript and performed the research. DM-A, RC-R, SP-Y, and JB-G performed the research. RM supervised the experimental design. JB supervised the experimental design as well as the analysis and interpretation of data. AV-F wrote the manuscript, conceived and designed the research and laboratory experiments, supervised the experimental design, and the analysis and interpretation of data. All authors read and approved the final manuscript.

## Conflict of Interest

RM was employed by company BioAssays Lab and UCM. The remaining authors declare that the research was conducted in the absence of any commercial or financial relationships that could be construed as a potential conflict of interest.
